# Reduced periprosthetic fracture rate when changing from a tapered polished stem to an anatomical stem for cemented hip arthroplasty: an observational prospective cohort study with a follow-up of 2 years

**DOI:** 10.1080/17453674.2019.1624339

**Published:** 2019-06-03

**Authors:** Jabbar Mohammed, Sebastian Mukka, Carl-Johan Hedbeck, Ghazi Chammout, Max Gordon, Olof Sköldenberg

**Affiliations:** a Department of Surgical and Perioperative Sciences, Umeå University;; b Department of Clinical Sciences, Division of Orthopedics, Karolinska Institutet at Danderyd Hospital, Stockholm, Sweden

## Abstract

Background and purpose — Straight collarless polished tapered stems have been linked to an increased risk for periprosthetic femur fractures in comparison with anatomically shaped stems, especially in elderly patients. Therefore, we evaluated the effect of an orthopedic department’s full transition from the use of a cemented collarless, polished, tapered stem to a cemented anatomic stem on the cumulative incidence of postoperative periprosthetic fracture (PPF).

Patients and methods — This prospective single-center cohort study comprises a consecutive series of 1,077 patients who underwent a cemented hip arthroplasty using either a collarless polished tapered stem (PTS group, n = 543) or an anatomic stem (AS group, n = 534). We assessed the incidence of PPF 2 years postoperatively and used a Cox regression model adjusted for age, sex, ASA class, cognitive impairment, BMI, diagnosis, and surgical approach for outcome analysis.

Results — Mean age at primary surgery was 82 years (49–102), 73% of the patients were female, and 75% underwent surgery for a femoral neck fracture. The PPF rate was lowered from 3.3% (n = 18) in the PTS group to 0.4% (n = 2) in the AS group. The overall complication rate was also lowered from 8.8% in the PTS group to 4.5% in the AS group. In the regression model only cognitive dysfunction (HR 3.8, 95% CI 1.4–10) and the type of stem (PTS vs AS, HR 0.1, CI 0.0–0.5) were correlated with outcome.

Interpretation — For elderly patients with poor bone quality use of cemented anatomic stems leads to a substantial reduction in periprosthetic fracture rate without increasing other complications.

A severe complication of hip arthroplasty is the periprosthetic femoral fracture (PPF), which is associated with increased mortality (Bhattacharyya et al. 2007, Young et al. 2008). The surgical treatment of PPF is demanding, with high complication and reoperation rates (Lindahl et al. 2005, 2006a, 2006b). Previous studies have reported differences between type of implant and the risk for PPF (Lindahl et al. 2005, Franklin and Malchau 2007, Palan et al. 2016).

The different designs of cemented femoral implants rely on different principles of fixation to the femur. The collarless polished tapered stems are designed to subside inside the cement mantle to achieve an even load-bearing and the matte composite-beam anatomical stems are designed to be fixed in the cement mantle. Straight collarless polished tapered stems have been linked to an increased risk for PPF in comparison with anatomically shaped stems, especially in elderly patients, with fracture as an indication for surgery (Lindahl et al. 2006b, Brodén et al. 2015, Mukka et al. 2016, Palan et al. 2016, Kristensen et al. 2018, Chatziagorou et al. 2019).

Based on these results our institution changed our standard femoral implant in 2014 for all cemented arthroplasty surgeries. We hypothesized that the transition from a straight, polished, tapered stem to an anatomic matte composite-beam stem would reduce the incidence of PPF and reoperations.  

## Patients and methods

### Study setting

This observational prospective cohort study was performed between 2012 and the beginning of 2018 (inclusion period 2012–2015) at the Orthopedic Department of Danderyd Hospital in Stockholm, Sweden. Danderyd Hospital is a university hospital affiliated with the Karolinska Institute and provides medical care to a catchment area of approximately 500,000 inhabitants.

### Study subjects

The study patients were identified from an ongoing prospective cohort study including a consecutive series of all hip arthroplasties performed at the Orthopedic Department of Danderyd Hospital. We included all patients operated between 2012 and 2015 with a cemented hip arthroplasty. During the first 2 years of the study (2012–2013) the control group (PTS) were recruited; in the case of bilateral surgeries during the inclusion period, only the 1st hip was included in the analysis.  

### Surgery

At our department, a cemented stem is used for both hemiarthroplasty (HA) and total hip arthroplasty (THA). For THA patients with degenerative joint disease and with a type A femur according to Dorr et al. (1993), an uncemented stem is often used. The choice of fixation method is ultimately up to the surgeon but the type of implants used is centralized and decided by the department. In 2014, a policy change was implemented whereby all cemented stems in HA and THA surgeries were changed from a polished tapered stem (PTS group) (CPT, Zimmer Inc., Warsaw, IN, USA) to an anatomic stem (AS group) (Lubinus SP2, Waldemar Link, Hamburg, Germany). In the fall of 2013, all surgeons were trained in the use of the new stem in seminars and with surgeries on saw-bones. Then, in January 2014, the polished, tapered stem was no longer available in the department and all cemented arthroplasty surgery was done using the new stem. The learning curve is thus included in the study.

The operations were performed either by a consultant orthopedic surgeon or by a registrar with assistance from a consultant. A modular, size 32 mm cobalt-chrome femoral head and an uncemented or a cemented acetabular component were used for patients operated with THA and a unipolar head for patients operated with HA.

### Surgical approach

According to surgeon preference, a standard posterolateral or a direct lateral approach was used. Based on a previous study from the same cohort of patients (Sköldenberg et al. 2010), the majority of patients with a femoral neck fracture are operated with a direct lateral approach whereas the posterolateral approach is preferred for patients with osteoarthritis.

### Peri- and postoperative prophylaxis

Antibiotic-loaded bone cement was used for all patients (Palacos with gentamicin, Heraues Medical GmbH, Wehrheim, Germany). Prophylactic antibiotics were administered 30 minutes preoperatively and twice more over 24 h postoperatively. Low-molecular-weight heparin was administered for 10–30 days postoperatively.

### Postoperative care

Patients were mobilized according to a standard physiotherapeutic program, and immediate full weight-bearing with the use of aids was encouraged. Patients who underwent surgery with a posterolateral approach were instructed to minimize flexion in combination with adduction and internal rotation for the first 3 months.

### Outcomes and data collection

Using the unique Swedish personal ID number, we collected data prospectively throughout the study period through a combination of a search of our in-hospital surgical and medical databases and regular follow-up visits. A digital case report form was used throughout the study. We also used the Swedish Hip Arthroplasty Register to identify any reoperations performed outside our hospital, but no such case was found.

All patients were followed up until 2 years after primary surgery or until death. The mean follow-up time was 20 months (median 24, 0–24 months) with no loss to follow-up.

The guidelines of the STROBE (STRrengthening the Reporting of OBbservational studies in Epidemiology) statement were followed.

### Variables

We collected data including stem type (PTS/AS) age, sex, cognitive dysfunction (no/yes, classified by the treating surgeon. Temporary confusion was not classified as cognitive dysfunction), ASA score, indication for surgery (primary osteoarthritis/other arthritic diseases (i.e. dysplasia, rheumatoid arthritis)/femoral neck fracture[FNF]/other fracture (i.e. trochanteric or acetabular), type of arthroplasty (THA/HA), surgical approach (posterolateral/direct lateral), and complications leading to reoperation including open surgery with revision of implants. Periprosthetic fractures were classified according to the Vancouver system (Brady et al. 2000) and the surgical treatment used in the reoperation (open reduction and internal fixation [ORIF]/stem revision). For patients with a PPF the radiographs were analyzed by OS, a senior consultant specialized in hip revision surgery.

The clinical and radiographic outcomes for the patients with PPF were evaluated by a combination of a medical chart review and radiographic analysis at follow-up visits as in a previously published study at our institution (Brodén et al. 2015). The outcome was graded as: “good” in patients with a radiographically healed fracture and no or little walking impairment, “intermediate” in patients with a healed fracture but impaired walking ability, and “poor” in patients with an unhealed fracture and a severely impaired walking ability.

### Sample size

Prior to the start of the study, a power analysis showed that a 5% significance level, and with 431 hips in each group, would give a power of 80% to detect a statistically significant difference in PPF rate with an assumed 3% fracture rate for the PTS group and a 0.5% rate for the AS group. Approximately 250–300 patients are operated yearly in the department with cemented hip arthroplasty and to achieve this sample we included all patients operated 2 years before, and 2 years after the change of implants.

### Statistics

For analysis of the primary outcome, we used Cox proportional hazards with follow-up time defined as time to death, reoperation, or end of follow-up (max. 2 years after surgery). Our main outcome variable was the occurrence of a PPF during the study period and we adjusted for exposure variable (PTS/AS), age, sex, ASA category, cognitive impairment, BMI, whether the indication was fracture or not, and surgical approach. Results are presented as hazard ratios (HRs) with 95% confidence intervals (CI).

The statistical analysis is based on the assumption that the studied observations are independent; therefore, no bilateral fractures were included. In patients with 2 fractures during the study period, only the 1st fracture was included.

All continuous variables were left as continuous but checked for non-linearity using ANOVA. We investigated the proportional hazards assumption using Grambsch and Therneau analysis of Schoenfeld residuals. All analyses were performed using R 3.5.2 (R Project for Statistical Computing, Vienna, Austria), using the rms package (v. 5.1-3) for survival modelling, knitr (v. 1.21) for reproducible research, ggplot2 for plots (v. 3.1.0) and Gmisc (v. 1.8) with Greg (v. 1.3) for table output.

### Ethics, funding, and potential conflicts of interest

The study was conducted in accordance with the ethical principles of the Helsinki Declaration and was approved by the Ethics Committee of Karolinska Institutet (entry number dnr 2013/285-31/2). According to the ethical permission, individual consent was not needed from the patients in this observational cohort. The study was funded by the regional agreement on medical training and clinical research (ALF) between Stockholm County Council and Karolinska Institutet and by a research grant from LINK. None of the funding bodies had any input into the data collection, analysis, or conclusions from the study. The authors declare no competing interests.  

## Results

### Study subjects and descriptive data

Of 2,007 hip arthroplasties performed at our institution during the inclusion period, 1,077 cemented arthroplasties in 1,077 patients were included in the final study cohort after exclusion of uncemented stems and 42 bilateral cases of cemented stems. The mean age at primary surgery was 82 years (49–102), 73% of the patients were female, and 75% underwent surgery for a femoral neck fracture. The baseline demographics were similar between the groups (Table 1). The 1- and 2-year mortality was 16% (n = 173) and 24% (n = 263), respectively with similar 2-year mortality between the groups (HR 1.1, CI 0.8–1.4).

**Table 1.** Characteristics of subject. Values are frequency (%) unless otherwise specified

	AS group	PTS group
Factor	n = 534	n = 543
Sex		
Male	135 (25)	156 (29)
Female	399 (75)	387 (71)
Age (years) (mean, SD)	82 (8.0)	82 (8.4)
ASA		
1–2	185 (35)	138 (25)
3–4	349 (65)	405 (75)
Height (cm)		
Mean (SD)	166 (9)	167 (9)
Missing	1 (0.2)	6 (1)
Weight (kg)		
Mean (SD)	67 (15)	68 (13)
Missing	1 (0.2)	3 (0.6)
BMI		
Mean (SD)	24 (4.5)	24 (4.1)
Missing	1 (0.2)	7 (1)
Cognitive dysfunction		
Yes	57 (11)	51 (9)
No	467 (87)	492 (91)
Missing	10 (2)	0 (0.0)
Indication for surgery		
Primary OA	124 (23)	94 (17)
Femoral neck fracture	383 (72)	421 (78)
Other arthritic	16 (3)	17 (3)
Other fracture	11 (2)	11 (2)
Type of hip arthroplasty)		
Total	248 (46)	211 (39)
Hemi	286 (54)	332 (61)
Surgical approach		
Direct lateral	354 (66)	411 (76)
Posterolateral	180 (34)	132 (24)

### Outcome data

**Figure 1.**
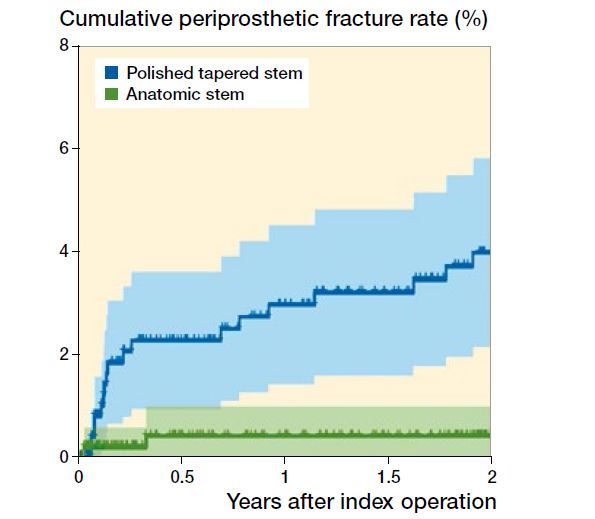
Cox regression of cumulative periprosthetic fracture rate after surgery adjusted for age, sex, cognitive dysfunction, BMI, indication for surgery, and surgical approach.

1.9% (n = 20) PPFs were identified during 2-year follow-up. The PPFs occurred at a median of 2 months (0.2–23) after primary surgery. The PPF rate was 3.3% (n = 18) in the PTS group and 0.4% (n = 2) in the AS group (Figure 1).

The fracture rate was higher for patients operated due to fracture in comparison with those with degenerative hip disease, 2.2% (n = 18) vs. 0.8% (n = 2). It was also generally higher for men, ASA category 3–4, cognitive dysfunction, posterolateral approach, and the use of the CPT stem (Table 2). However, in the multivariable Cox proportional hazard regression only cognitive dysfunction (HR 3.8, CI 1.4–10) and the type of stem (PTS as denominator, HR 0.1, CI 0.0–0.5) were statistically significant.

**Table 2.** Cox proportional hazard regression crude and adjusted models Association with periprosthetic fracture presented as Hazard ratio (HR)

Variable	Total	PPF n (%)	Crude HR (95%Cl)	Adjusted HR (95%C)
Age, mean (SD)	82 (8)		1.0 (0.9–1.1)	1.0 (0.9–1.0)
Sex				
Male	291	9 (3.1)	1.0 ref.	1.0 ref.
Female	786	11 (1.4)	0.4 (0.2–1.0)	0.4 (0.1–0.9)
ASA category				
1–2	323	2 (0.6)	1.0 ref.	1.0 ref.
3–4	754	18 (2.4)	4.5 (1.0–19)	3.0 (0.7–14)
Cognitive dysfunction				
No	959	14 (1.5)	1.0 ref.	1.0 ref.
Yes	108	6 (5.6)	4.4 (1.7–12)	3.8 (1.4–10)
BMI, mean (SD)	24 (4)		0.9 (0.8–1.0)	0.9 (0.8–1.0)
Indication for surgery				
Degenerative hip	251	2 (0.8)	1.0 ref.	1.0 ref.
Fracture	826	18 (2.2)	3.2 (0.7–14)	3.8 (0.6–24)
Approach				
Direct lateral	765	15 (2.0)	1.0 ref.	1.0 ref.
Posterolateral	312	5 (1.6)	0.7 (0.3–2.0)	3.2 (0.9–12)
Group				
PTS	534	2 (0.4)	1.0 ref.	1.0 ref.
AS	543	18 (3.3)	0.1 (0.0–0.5)	0.1 (0.0–0.5)

The overall complication rate including PPFs was 6.7% with 8.8% (n = 48) in the PTS group and 4.5% (n = 24) in the AS group (p = 0.004). In the PTS group, PPF was the most common complication (3.3%) followed by dislocation (2.6%) and periprosthetic joint infection (2.2%). In the AS group, periprosthetic joint infection was the most common reason for revision surgery (1.7%), followed by dislocation (1.1%) and PPF (0.4%).

**Table:** 

Values are percentage at risk					
	Years after index operation				
	0	0.5	1	1.5	2
PTS	100	83	78	74	69
AS	100	85	81	77	67

### Periprosthetic fractures

70% of PPFs were Vancouver type B2 (n = 12) and type C fractures (n = 2); none of the hips had any radiographic sign of loosening of the stem or periprosthetic osteolysis before fracture. All were sustained through low-energy falls. 13 of the PPFs were treated with stem revision. 12 of 20 PPFs had a good outcome according to the previous definition (Table 3). 

**Table 3.** Periprosthetic fractures, surgical treatment and surgical outcome

Factor	PTS	AS
Vancouver classification		
A	2	1
B1	3	0
B2	12	0
B3	0	0
C	1	1
Surgical treatment:		
Open reduction and internal fixation	6	1
Stem revision	12^a^	1
Surgical outcome		
Good	10	2
Intermediate	4	0
Poor	4	0

a 10 of 12 Vancouver B2 fractures were treated with stem revision.

## Discussion

In this prospective, observational cohort study on an elderly cohort of patients comparing a collarless polished tapered stem with an anatomic stem, we have shown that it is possible to reduce the periprosthetic fracture rate dramatically for an orthopedic department by changing the standard implant used for hip arthroplasty patients. As we included all patients during the study period, this study therefore includes the whole department’s learning curve with the new stem.

The main strength of our study is the prospective design with an isolated change of stem implant without a change in catchment area, surgeons, indication for surgery, or other routines at an orthopedic department. The completeness of data on the incidence of surgically treated PPF and the homogeneity of implant choice are other strengths.

The limitations of this study are the lack of randomization for stem type, leading to a risk of confounding variables in spite of the fact that we that used adjusted regression models. Based on our department’s indication for surgery, these results only apply and are limited to an elderly cohort of patients with degenerative hip disease and femoral neck fracture. The limited sample size and the short follow-up time are counterbalanced by the study design. There is still a need for prospective cohort studies due to risk of under-reporting reoperations to the Swedish hip arthroplasty registry of those PPFs treated with open reduction and internal fixation without change of the implant (Thien et al. 2014, Swedish hip arthroplasty registry. Annual report 2017).

In concordance with previous studies from our department, we found a high incidence of early PPFs associated with the CPT stem (Brodén et al. 2015, Mukka et al. 2016). It seems that the CPT stem, designed to subside in the cement mantle with axial load, acts as a wedge breaking the femur after a direct hip contusion—a concept discussed by Sarvilinna et al. (2004). The tapered stem generates a stress riser, which in turn splits the femur into complex periprosthetic Vancouver B fractures. We found 12 of the 18 PPFs in the proximity of the CPT stem, generating Vancouver B2 fractures necessitating stem revision using longer cemented or uncemented implants.

The standard length (130 mm) of the CPT stem is shorter than the most commonly used version in Sweden of the Lubinus SP2 stem (150 mm). Longer cemented stems anchored distally in harder diaphyseal bone might conceptually reduce this risk further. This is supported by mechanical studies showing that shorter stems have a decreased resistance to torque forces (Bishop et al. 2010, Morishima et al. 2014). However, the shorter 130 mm Lubinus SP2 stem has shown a good long-term outcome without any increased risk for PPF, in a younger population treated mainly for osteoarthritis (Prins et al. 2014). The anatomical shape of the Lubinus SP2 provides a prerequisite for a homogenic cement mantle around the prosthesis which, in turn, reduces the risk of contact between the tip of the prosthesis and cortical bone.

Several studies have cited risk factors predisposing to PPF such as sex, advanced age, ASA 3 or 4, osteoporosis, surgical approach, and type of implant (Sarvilinna et al. 2004, Franklin and Malchau 2007, Cook et al. 2008, Jasvinder et al. 2013). We did not find any association between sex and the risk for PPF. Inngul and Enocson (2015) described an increased risk for PPF in men while other studies found a higher proportion of PPF among women (Franklin and Malchau 2007, Sheth et al. 2013).

Age has frequently been proposed as a risk factor for PPF; elderly patients are at a higher risk for osteoporosis and frequent falls, which in turn predisposes to PPF (Franklin and Malchau 2007).

Cook et al. (2008) described lower risk of fracture for patients below the age of 70 and the highest among those above 80 years of age. Rheumatoid arthritis is a risk factor for PPF, because of a decrease in bone mineral density (Haddad et al. 1999). We could not confirm ASA class 3 or higher as a risk factor for PPF (Singh et al. 2013). In concordance with our findings, Sarvilinna et al. (2004) and Jasvinder et al. (2013) did not find any association between BMI and PPFs after THR.

It has been suggested that the surgical approach used could alter the rate of PPF. A higher risk has been proposed for the direct lateral approaches due to a higher incidence of anteroposterior malalignment in the sagittal view (Garellick et al. 1999, Lindahl et al. 2006a). A very recently published registry-based study by Chatziagorou et al. (2019) found an increased risk associated with the posterior approach. The differences in implantation of the stems might affect the loading and behavior of the implant and thus alter the risk for PPF. The possible influence of surgical approach on the risk for PPF needs to be addressed in future studies.

In summary, in this prospective cohort study of a change of stem implant at an orthopedic department, the use of an anatomic stem as compared with a collarless polished tapered stem resulted in a substantially lower rate of periprosthetic femoral fractures without increasing other complications such as dislocation. In hospitals where elderly patients with poor bone quality are operated, the switch to use of cemented anatomic stems is to be recommended.

JM analyzed the data and wrote the manuscript. SM supervised JM, analyzed the data, and wrote the manuscript. GC and CJH operated patients and wrote the manuscript. MG analyzed data and reviewed the manuscript. OS initiated the study, collected data, supervised JM, operated patients, and wrote the manuscript. 

*Acta* thanks Anders Enocson and Geert Meermans for help with peer review of this study.
